# Characterization of the Human Sigma-1 Receptor Chaperone Domain Structure and Binding Immunoglobulin Protein (BiP) Interactions*

**DOI:** 10.1074/jbc.M113.450379

**Published:** 2013-06-12

**Authors:** Jose Luis Ortega-Roldan, Felipe Ossa, Jason R. Schnell

**Affiliations:** From the Department of Biochemistry, University of Oxford, Oxford OX1 3QU, United Kingdom

**Keywords:** Endoplasmic Reticulum Stress, Membrane Proteins, NMR, Signaling, Structural Biology, BiP, Sigma-1 Receptor

## Abstract

The sigma-1 receptor (S1R) is a ligand-regulated membrane protein chaperone involved in the ER stress response. S1R activity is implicated in diseases of the central nervous system including amnesia, schizophrenia, depression, Alzheimer disease, and addiction. S1R has been shown previously to regulate the Hsp70 binding immunoglobulin protein (BiP) and the inositol triphosphate receptor calcium channel through a C-terminal domain. We have developed methods for bacterial expression and reconstitution of the chaperone domain of human S1R into detergent micelles that enable its study by solution NMR spectroscopy. The chaperone domain is found to contain a helix at the N terminus followed by a largely dynamic region and a structured, helical C-terminal region that encompasses a membrane associated domain containing four helices. The helical region at residues ∼198–206 is strongly amphipathic and proposed to anchor the chaperone domain to micelles and membranes. Three of the helices in the C-terminal region closely correspond to previously identified cholesterol and drug recognition sites. In addition, it is shown that the chaperone domain interacts with full-length BiP or the isolated nucleotide binding domain of BiP, but not the substrate binding domain, suggesting that the nucleotide binding domain is sufficient for S1R interactions.

## Teaser

**Background:** Sigma-1 receptor is a ligand-regulated membrane protein chaperone involved in BiP regulation and the ER stress response.

**Results:** The chaperone domain of human sigma-1 receptor is mostly helical with short extended regions.

**Conclusion:** Regions of the sigma-1 receptor chaperone domain implicated in ligand and cholesterol binding can be mapped to separate helices.

**Significance:** A structural framework for delineating sigma-1 receptor BiP and ligand interactions is presented.

## Introduction

The sigma-1 receptor (S1R)^3^ is a ligand-regulated membrane protein chaperone involved in the ER stress response and interorganelle communication (1–3). S1R is localized to mitochondria-associated ER membranes (4, 5), which are sites for regulation of mitochondrial bioenergetics via ER calcium release (6). S1R is expressed primarily in cerebral cortex, hippocampus, and cerebellum Purkinje cells (7, 8), and has been proposed as a target for treatment of central nervous system diseases, including amnesia, pain, schizophrenia, clinical depression, Alzheimer disease, stroke, and addiction (9, 10). S1R activity is modulated by *N*,*N*-dimethyltryptamine (11), progesterone (12), and sphingosine (13). In addition, S1R is regulated by a large number of exogenous small molecules, including opiates, antipsychotics, antidepressants, antihistamines, phencyclidine-like compounds, β-adrenergic receptor ligands, and cocaine (reviewed in Ref. 10).

Activated S1R dissociates ankyrin from the inositol triphosphate receptor (3, 14), which results in calcium release at the mitochondria-associated ER membrane that is efficiently taken up by mitochondria to increase energy production. S1R appears also to have other roles in the stress response. ER calcium depletion or agonist binding dissociates S1R from the Hsp70 protein BiP, resulting in activation of protein chaperoning activity in both BiP and S1R (1). Chaperone activity and BiP binding (1) and the interaction of S1R with inositol triphosphate receptor (14) have been localized to the C-terminal domain of S1R, and truncation of the C terminus leads to dysfunction in mitochondrial stress response (15).

**FIGURE 1.**
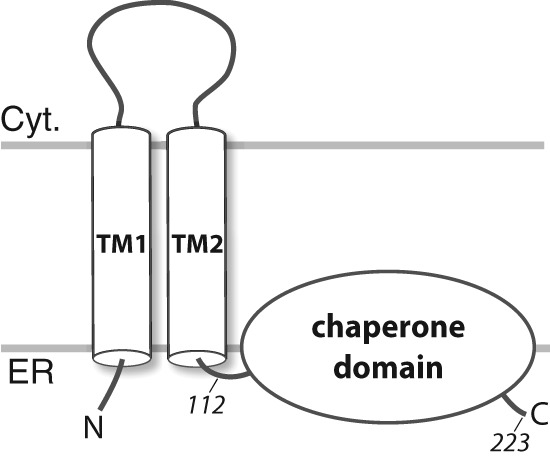
**Full-length S1R topology schematic showing two predicted transmembrane helices (TM1 and TM2) and the membrane-associated domain.** The human S1R(cd) construct studied here contains residues 112–223 that include a predicted disordered region (residues ∼134–167 (40)) and a predicted membrane-associated domain (residues ∼176–204 (47)). The N and C termini and the approximate positions of residues 112 and 223 (the C-terminal residue) are indicated. The membrane topology is based on Refs. 1 and 48. *TM1*, transmembrane domain 1; *TM2*, transmembrane domain 2; *Cyt.*, cytosol.

The chaperone domain is C-terminal to two putative transmembrane domains (residues 11–29 and 80–100) but contains a predicted membrane associated region (residues ∼176–204) containing two cholesterol recognition motifs (CRM) (Fig. 1) (16, 17). We have studied the S1R chaperone domain (residues 112–223; S1R(cd)) reconstituted into detergent micelles by solution NMR. S1R(cd) solubilized in dodecylphosphocholine (DPC) adopts a conformation competent to bind BiP in a calcium-dependent manner. Analysis of the structure and dynamics indicates that S1R(cd) contains five helices and at least two short extended regions. Three of the helices in the C-terminal membrane-associated domain correspond closely to regions previously implicated in cholesterol and drug interactions.

## EXPERIMENTAL PROCEDURES

### 

#### 

##### Protein Sample Production

An ACA-free gene construct (GeneArt) containing a (His)_6_-tag, a factor Xa cleavage site, and residues 112–223 of human S1R was subcloned into the pCOLD-I vector (Takara) and confirmed by sequencing. The N-terminal sequence preceding residues 112–223 of S1R was MNHKVHHHHHHIEGRHM. The S1R(cd) plasmid and a pMazF plasmid containing the gene for the RNA interferase MazF (Takara) were transformed into C43(DE3) cells. Transformed cells were grown to an OD of 0.8–0.9, cold shocked on ice, and incubated for 45 min at 15 °C. Cells were pelleted and washed with M9 salt solution, spun again, and resuspended into isotopically labeled medium with a 10-fold condensation. Cells were then incubated for a further 45–60 min at 15 °C before induction with 2 mm isopropyl 1-thio-β-d-galactopyranoside. Expression proceeded for 16 h at 15 °C. Both membranes and inclusion bodies were collected with a 40,000 rpm spin and incubated overnight in a solution containing 6 m guanidine, 200 mm NaCl, 1% Triton X-100, and 20 mm Tris, pH 8.0. S1R(cd) was separated by nickel affinity chromatography and dialyzed against water to remove guanidine. The precipitated protein was resolubilized in hexafluoro-2-propanol and purified by HPLC on a C3 reverse phase column over a gradient from buffer A (95% water, 5% acetonitrile, 0.1% trifluoroacetic acid) to buffer B (57% 2-propanol, 38% acetonitrile, 5% water, 0.1% trifluoroacetic acid). Fractions containing S1R(cd) were pooled and lyophilized. Yields of pure S1R(cd) from triple-labeled medium were between 8 and 10 mg per liter of labeled medium.

Polyhistidine-tagged human BiP (residues 26–654; “full length”), the isolated nucleotide binding domain (NBD; residues 26–410), and the isolated substrate binding domain (SBD; residues 418–638) of BiP (Herwig Schüler, Karolinska Institutet) were expressed in BL21(DE3) cells at 18 °C according to a previously published protocol (18). An ammonium sulfate precipitation was introduced as an initial purification step. The proteins were further purified by nickel chromatography, anion exchange chromatography using a Q-Sepharose column, and gel filtration using a Superdex200 column.

##### S1R(cd) Sample Preparation

S1R(cd) was reconstituted with detergent micelles for NMR measurements from thin films of protein and DPC that were solubilized in hexafluoro-2-propanol and dried down under a nitrogen stream (19). The film was resolubilized in urea and reconstituted by dialysis against NMR buffer containing 20 mm HEPES, pH 6.5. Based on integration of peaks in ^1^H one-dimensional spectra calibrated against samples with known detergent concentrations, the final amount of DPC in S1R(cd) NMR samples was ∼30 mm. Where indicated, certain samples of S1R(cd) were run over gel filtration rather than dialyzed to reduce the DPC concentration to 5 mm. Final protein concentrations for all NMR samples were ∼250 μm.

##### NMR Spectroscopy

All NMR experiments were collected at 37 °C at field strengths of 500, 600, 750, or 950 MHz (^1^H). The 750 and 950 MHz spectrometers were equipped with room temperature probes (home-built), and the 500 and 600 MHz spectrometers were equipped with cryogen-cooled probes (Bruker). NMR spectra were processed using NMRPipe (20) and analyzed using NMRView or CARA. Backbone ^1^H_N_, ^15^N, and ^13^Cα, and side chain ^13^Cβ assignments were obtained from triple resonance HNCA, CBCA(CO)NH, HNCACB, and HNCO spectra on ^15^N, ^13^C, and ^2^H-labeled S1R(cd). Assignments were confirmed and extended to sidechain protons with ^15^N-edited NOESY (90- or 180-ms mixing times at 950 or 600 MHz, respectively) and ^15^N-edited TOCSY spectra (40-ms mixing time; 600 MHz). ^15^N R_1_, R_2_, and ^1^H-^15^N heteronuclear NOE experiments were collected on a 0.27 mm ^15^N-labeled S1R(cd) sample at 600 MHz using the following relaxation delays: 4, 350, and 480 ms (T_1_) and 0, 40, and 60 ms (T_2_).

##### Water-soluble Paramagnetic Studies

Mn^2+^EDDA^2−^ was prepared as described (21). ^1^H,^15^N HSQCs were recorded before and after addition of 1 mm Mn^2+^EDDA^2−^. The accessibility to water was taken to be inversely proportional to the ratio of cross-peak intensity in the absence (*I*) and presence (*I*_o_) of Mn^2+^EDDA^2−^.

##### BiP Interactions

BiP titrations were carried out by preparing concentrated stocks of BiP constructs in 100 mm NaCl, 30 mm DPC, 5 mm 2-mercaptoethanol, and 20 mm HEPES, pH 6.5. Isotope-labeled samples of S1R(cd) in identical conditions were titrated with BiP, and ^1^H,^15^N SOFAST-HMQC spectra (22) were collected to monitor changes in cross-peak intensities and chemical shifts.

##### Circular Dichroism

CD spectra were collected on a Jasco J-815 circular dichroism spectropolarimeter by collecting spectra from 190–250 nm with 10 accumulations (far UV) or 250–350 nm with 10 accumulations. Samples for CD contained 17.5 μm (far UV) and 240 μm (near UV) S1R(cd) in 5 mm DPC and 20 mm potassium phosphate at pH 6.5.

## RESULTS

### 

#### 

##### S1R(cd) Sample Production and Structural Overview

**FIGURE 2.**
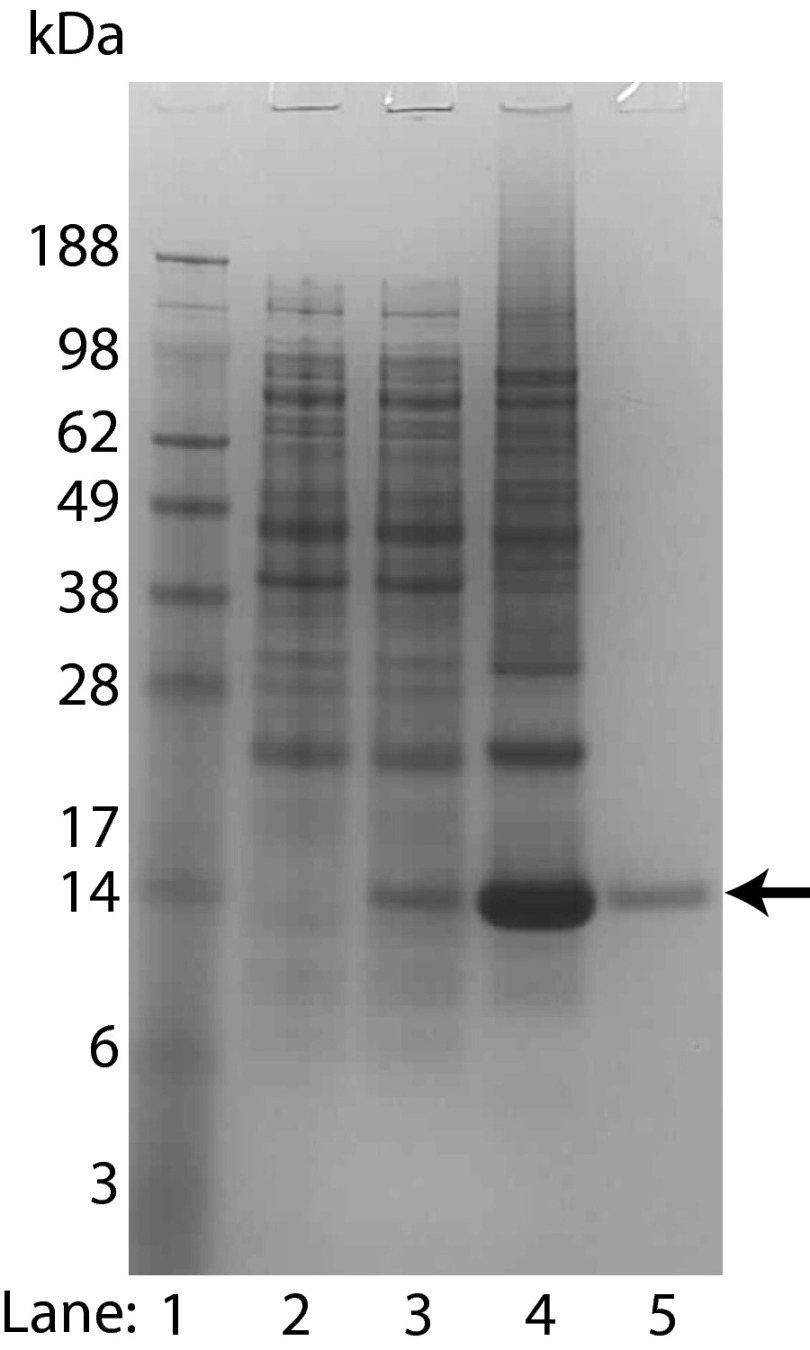
**SDS-PAGE stained with Coomassie Blue showing bacterial production of triple-labeled (^2^H, ^13^C, and ^15^N) S1R(cd) using the single protein production system.** The S1R(cd) (theoretical mass of 14.7 kDa) band is indicated by an *arrow. Lane 1*, molecular weight standard; *lane 2*, whole cell lysate before induction; *lane 3*, whole cell lysate after induction in minimal medium for 16 h at 15 °C; *lane 4*, precipitated protein after elution from nickel column; and *lane 5*, HPLC-purified protein.

**FIGURE 3.**
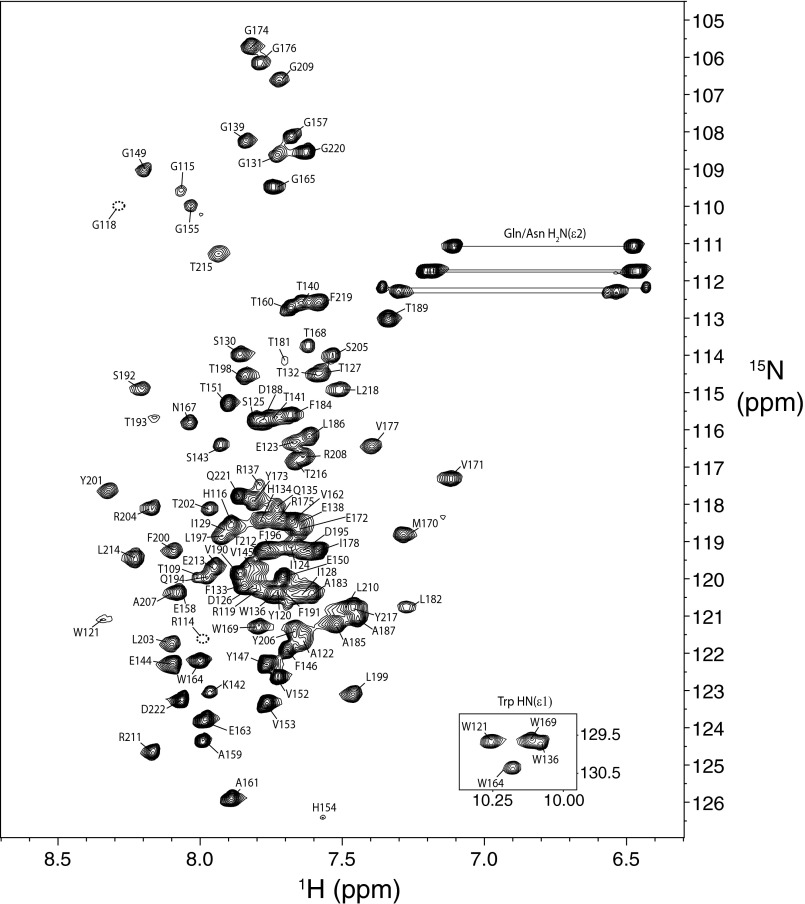
**^1^H,^15^N HSQC spectrum (600 MHz, ^1^H) of S1R(cd) in 5 mm DPC at 37 °C.** Backbone amide resonance assignments are indicated. The positions of the cross-peaks corresponding to residues Arg-114 and Gly-118, which are weak, are indicated with *dashed line circles*. The five cross-peak pairs expected from asparagine and glutamine H_2_N(ϵ2) groups of the S1R(cd) construct are indicated with *horizontal lines. Inset*, the four tryptophan HN(ϵ1) cross-peaks.

An S1R construct (S1R(cd)) containing the residues following the second putative transmembrane domain of S1R was produced with an N-terminal (His)_6_ tag using the single protein production approach (23) and purified to homogeneity (Fig. 2). Attempts to reconstitute S1R(cd) into aqueous solution in the absence of lipids or detergent resulted in protein aggregation. Therefore, S1R(cd) was reconstituted in the presence of DPC micelles. The resulting sample yielded homogenous NMR spectra permitting acquisition of high resolution data (Fig. 3).

**FIGURE 4.**
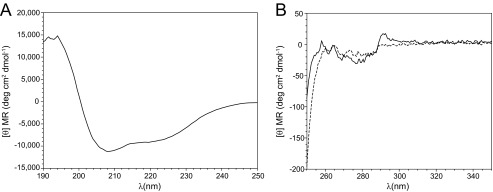
**Circular dichroism spectra of S1R(cd) in 5 mm DPC and 20 mm HEPES, pH 6.5, plotted as the mean residue molar ellipticity ([θ] *MR*).** *A*, far UV spectrum of S1R(cd) (17.5 μm) at 37 °C. *B*, near UV spectra of S1R(cd) (240 μm) at 37 °C (*solid line*) and 95 °C (*dashed line*).

The overall secondary and tertiary structure of S1R(cd) was probed with CD. Far UV CD of S1R(cd) in 5 mm DPC exhibited minima at 208 nm and ∼220 nm (Fig. 4*A*). A lack of a well defined minimum at 222 nm suggested the presence also of a small amount of β-strand structure. Tertiary structure was assessed by CD at near UV wavelengths (Fig. 4*B*). A negative peak at 280 nm and a positive peak at ∼290 nm indicated the presence of tertiary structure. To test whether the tertiary structure could be disrupted at high temperature, a second spectrum was collected at 95 °C. At 95 °C, the 290-nm peak disappears and the 280-nm peak is decreased, indicating unfolding at high temperature.

**FIGURE 5.**
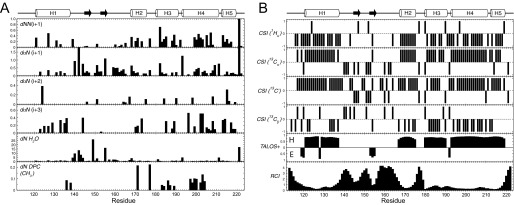
*A*, summary of NOE cross-peaks to backbone amide protons. Amide proton to amide proton NOEs (*dNN*(*i*+*1*)), and amide proton to α-proton NOEs (*d*α*N*(*i*+*1*), *d*α*N*(*i*+*2*), and *d*α*N(i*+*3*)), as well as cross-peaks at the position of the water proton (*dN H*_2_*O*), were measured in a three-dimensional ^15^N-edited NOESY with 180-ms mixing time at 600 MHz (^1^H) on an ^15^N-labeled S1R(cd) in 5 mm DPC. Amide proton to DPC methyl proton NOEs (*dN DPC* (*CH*_3_)) were obtained from a three-dimensional ^15^N-edited NOESY with 90 ms of mixing time at 950 MHz (^1^H) on an ^15^N- and ^2^H-labeled (∼70%) S1R(cd) in 30 mm DPC. The *x* axis (arbitrary units) is the cross-peak intensity normalized to that of the diagonal. No attempt was made to deconvolute overlapped cross-peaks. *B*, the chemical shift indices (*CSI*) of ^1^Hα, ^13^Cα, ^13^C′, and ^13^Cβ are shown in the *top four panels*. In *panels five* and *six* are shown the secondary structure and random coil index derived from chemical shift analysis using TALOS+ (49) and the method of Berjanskii *et al.* (50), respectively. For the TALOS+ plot, values *above* or *below the line* indicated that the calculated φ/ψ values correspond to helical (*H*) or extended conformation (*E*), respectively. Shown schematically at the *top* are the S1R(cd) residues determined to be helical (*cylinders*) or extended (*arrow*) (see main text for full description of secondary structure assignment).

Conventional amide proton-based NMR experiments enabled backbone resonance assignment of 104 of the 107 nonproline residues of S1R(cd) (Fig. 3). Many of the amide proton NOESY strips for residues ∼140 to 160 contained cross-peaks at the water proton chemical shift indicating chemical exchange, and few medium range NOEs (*i.e. d*α*N*(*i* + *3*)) (Fig. 5*A*). In contrast, NOE strips for residues ∼120–140 and ∼160–220 exhibited extensive short and medium range NOEs.

Secondary chemical shift analysis was used to facilitate determination of S1R(cd) secondary structure (Fig. 5*B*). Chemical shift indexing of individual nuclei and TALOS+ analysis predicted helices at residues ∼121–137, ∼167–175, ∼180–189, and ∼193–219. Although chemical shifts predicted a continuous helix from residues ∼193–219, a large increase in the measured rate of exchange of the amide protons of residues 211–213 with water indicated disruption of the helical hydrogen bonding network and a break in this helix (see below). In addition, secondary chemical shifts predicted a φ/ψ angle for Ile-128 that corresponded to an extended conformation, suggesting that helix 1 may also be disrupted. However, there were no corresponding increases in the amide exchange rate or dynamics for this residue (see below).

Regions of extended structure could be determined with less confidence than helical regions, although an extended conformation is likely for residues ∼145–147 and ∼153–155. Chemical shift indices analysis and intense *daN*(*i* + *1*) NOEs suggested the possibility of a third β-strand in residues ∼160–162, but the RCI indicated a high degree of flexibility in these residues.

##### Backbone Dynamics of S1R(cd)

**FIGURE 6.**
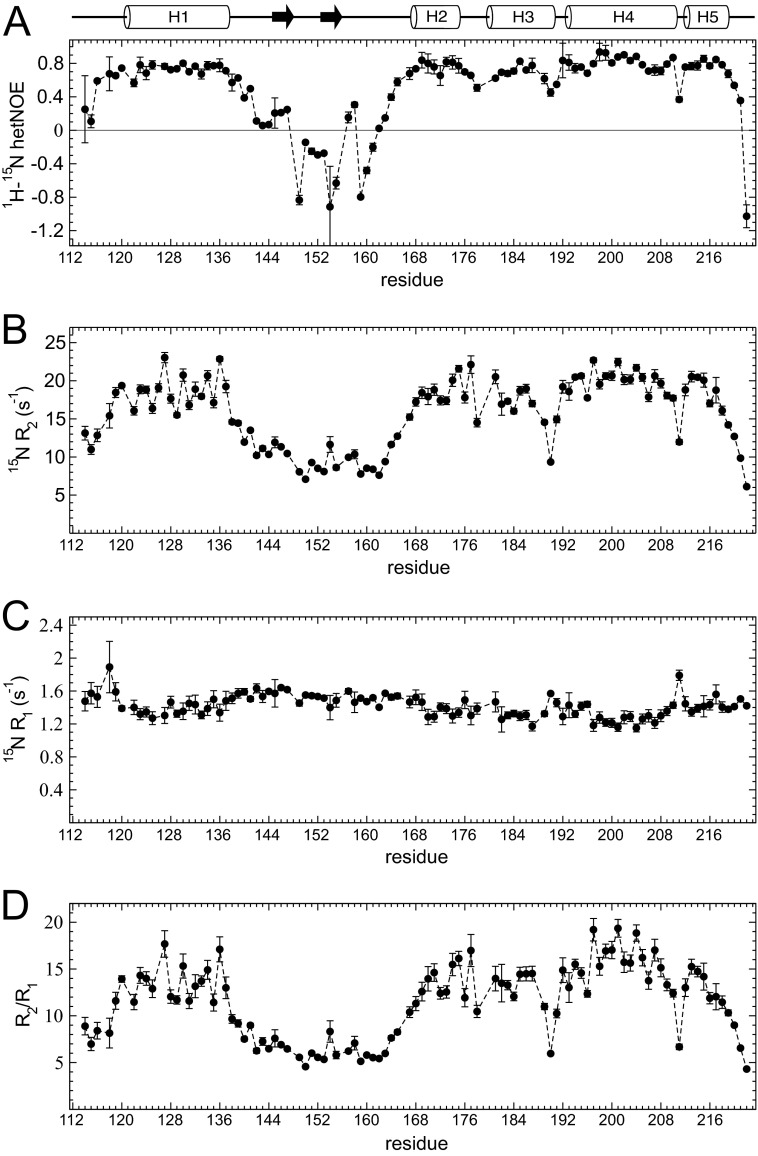
**Relaxation properties of S1R(cd).** *A*, ^1^H-^15^N heteronuclear NOEs (*het-NOE*); *B*, ^15^N transverse relaxation rates (^15^N R_2_); *C*, ^15^N longitudinal relaxation rates (^15^N R_1_); and *D*, ratio of ^15^N R_2_ and R_1_, as a function of residue number for S1R(cd) at 600 MHz (^1^H).

Backbone amide dynamics of S1R(cd) were probed by measuring ^15^N transverse (R_2_) and longitudinal (R_1_) relaxation rates and ^1^H-^15^N heteronuclear NOEs (Fig. 6). Small ^15^N R_2_ values and negative or small heteronuclear NOEs were found throughout the region of residues 142–163, consistent with the low amount of structure in this region, although increased heteronuclear NOEs in residues 145–147 correlated with the chemical shift-based prediction that these residues are in a stable extended conformation. In addition, the relaxation properties of some of the interhelical regions at the C terminus exhibited decreased heteronuclear NOEs and ^15^N R_2_ relaxation rates, indicating increased flexibility, particularly in Ile-178 between helices 2 and 3, Val-190 between helices 3 and 4, and Arg-211 between helices 4 and 5.

R_2_/R_1_ ratios correlate with the effective rotational correlation time (24) and are plotted in Fig. 6*D* for S1R(cd). The average R_2_/R_1_ value for helical residues was 14.0 ± 2.2, which corresponds to a rotational correlation time (τ_c_) of ∼11.6ns. This τ_c_ is nearly twice that expected for a 14.7-kDa protein at 37 °C (∼6.1 ns), suggesting that the protein is tightly associated with a detergent micelle. Although residues in helices 1, 2, 3, and 5 exhibited similar mean τ_c_ values (10.9–11.5 ns), residues in helix 4 had a mean τ_c_ value of 12.4 ns. Although effects from rotational anisotropy or contributions to the R_2_ from conformational exchange could not be ruled out, the higher τ_c_ value for helix 4 was consistent with this helix anchoring S1R(cd) to the detergent micelle (see below).

##### Water and Micelle Interactions of S1R(cd)

**FIGURE 7.**
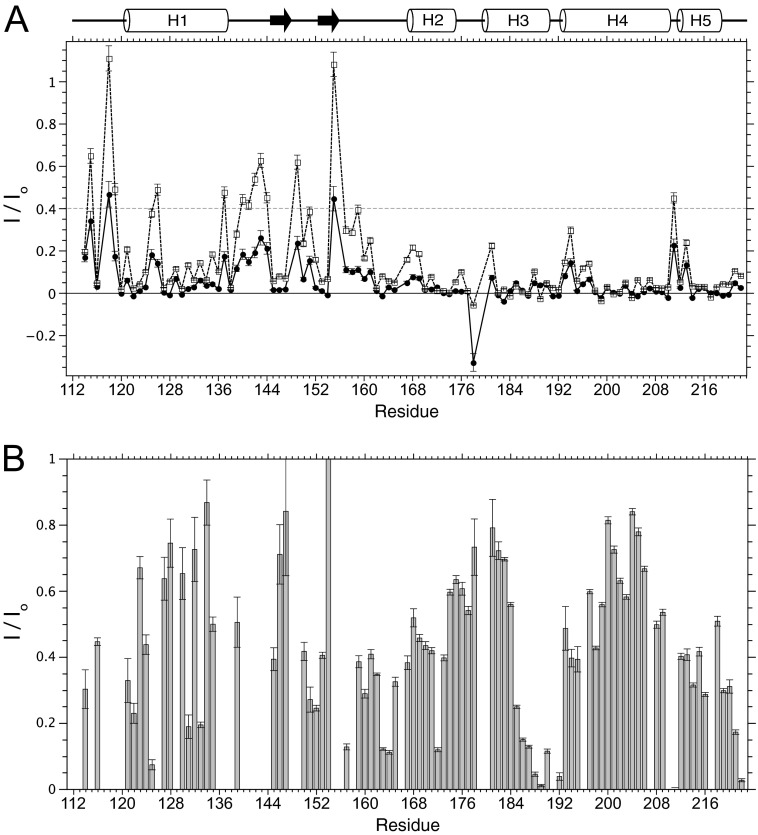
**Summary of water interactions with S1R(cd).** *A*, backbone amide proton exchange of S1R(cd) with water measured using CLEANEX experiments (25). Water proton exchange is shown as the ratio of the peak volume in CLEANEX experiments using 10-ms (*filled circles*) or 30-ms (*open squares*) mixing times to the peak volume in a fast HSQC (600 MHz ^1^H). Measurement error has been estimated from the spectral noise. *B*, resonance broadening after addition of the water-soluble paramagnetic agent Mn^2+^EDDA^2−^. A decreased ratio of the peak intensity after (*I*) and before (*I*_o_) addition of Mn^2+^EDDA^2−^ indicates increased water accessibility. Measurement error has been estimated from the spectral noise. Data for resonances that exhibit high water exchange rates as indicated by CLEANEX ratios of *I*/*I*_o_ > 0.4 (*dashed line* in *A*) were excluded from the analysis due to potential artifacts in cross-peak intensities due to changes in relaxation properties.

Based on observation of cross-peaks at the water proton resonance frequency in the NOESY, a large number of backbone amides within the region of residues 140–160 had apparent amide proton chemical exchange with water. Therefore, the rates of backbone amide exchange with water were measured using CLEANEX experiments (Fig. 7*A*) (25). In the region between helices 1 and 2, residues 137, 139–144, 149–151, and ∼155–161 had the largest exchange rates. Exchange rates in the predicted extended regions (residues 145–147 and 153–155) were low, suggesting the presence of β-sheet structure. Increases in exchange were also observed in the interhelical regions in the C-terminal half of S1R(cd) and helped to assign breaks in the helices.

Low amide proton exchange rates may be due to hydrogen bonding and/or inaccessibility to bulk water. Therefore, we measured line-broadening effects from the water-soluble paramagnetic agent Mn^2+^EDDA^2−^. Broadening of many resonances corresponding to the N-terminal half of S1R(cd) was observed, indicating few continuous regions of protection from water. In contrast, the two segments of residues ∼174–184 and ∼197–206 showed relatively high levels of protection (*I*/*I*_o_ > ∼0.5), indicating interactions with the detergent micelle and/or other regions of the protein.

Regions of interactions of S1R(cd) with the detergent micelle were assessed from an ^15^N-edited NOESY (90 ms) recorded on an ^15^N-labeled and partially deuterated sample at high field (950 MHz) to resolve cross-peak overlaps and reduce spin diffusion (Fig. 5*A*). The largest clusters of residues with NOEs to detergent occurred in residues 183–189 in helix 3 and residues 197–204 in helix 4. Helix 4 was also strongly protected from the water-soluble paramagnetic agent. The region ∼198–206 forms an amphipathic helix, with residues Leu-199, Phe-200, Leu-203, and Tyr-206 forming the hydrophobic face (26).

##### S1R(cd) Interactions with BiP

**FIGURE 8.**
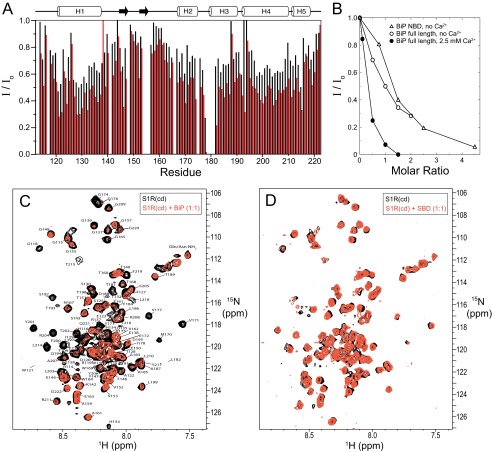
**S1R(cd) interactions with BiP.** *A*, backbone amide cross-peak intensities are plotted as a function of residue number after addition of 0.5 (*black bars*) and 1.0 (*red bars*) molar equivalents of full-length BiP (no calcium). The intensities are normalized to the cross-peak intensities in the absence of BiP. *B*, the normalized average backbone amide cross-peak intensity of S1R(cd) as a function of the molar ratio of BiP NBD (no calcium) or full-length BiP to S1R(cd) in the absence or presence of 2.5 mm calcium chloride. The intensity average is for only the helical regions. *C*, spectral overlays of ^15^N-labeled S1R(cd) with (*red*) or without (*black*) full-length BiP (1:1) in the presence of 2.5 mm calcium. *D*, spectral overlays of ^15^N-labeled S1R(cd) with (*red*) and without (*black*) addition of the BiP SBD (1:1) in the presence of 2.5 mm calcium.

Previous work by Hayashi *et al.* (1) showed that S1R residues 116–223 are sufficient for binding to the Hsp70 protein BiP. Therefore, the NMR spectrum of ^15^N-labeled S1R(cd) was monitored as a function of unlabeled BiP concentration (Fig. 8). The S1R(cd) cross-peak intensities decreased in a BiP concentration-dependent manner, suggesting that a BiP·S1R(cd) complex was formed. After several hours, a white precipitate formed, indicating that the complex was unstable in solution. By contrast, BiP alone in the titration buffer (containing 30 mm DPC) was more stable. It was speculated that binding of the S1R(cd)·micelle complex to BiP may increase the local concentration of DPC to destabilize BiP.

The changes in ^1^H-^15^N cross-peak intensities upon addition of full-length BiP were plotted as a function of residue number at molar ratios of 0.5 and 1 ([BiP]/[S1R(cd)]) (Fig. 8*A*). Decreases in cross-peak intensities were higher for residues in the structured helical regions and smaller in the flexibly disordered regions at the N and C termini and residues ∼140–160, suggesting that these disordered residues retained some flexibility in the complex.

BiP chaperone (27) and ATPase (1) activity has been shown to be inhibited by calcium, and the interaction between BiP and S1R is proposed also to be modulated by calcium under physiological conditions, with the presence of calcium leading to increased association and inactivation of S1R and BiP (1). Therefore, a second titration of full-length BiP against S1R(cd) was carried out in the presence of 2.5 mm calcium chloride. A comparison of the cross-peak intensities for helical residues as a function of BiP indicates an increased association of S1R(cd) for full-length BiP in the presence of calcium (Fig. 8*B*). No chemical shift changes were observed upon addition of calcium to S1R(cd) alone, consistent with a calcium binding site on BiP (18).

To test whether the NBD of BiP was sufficient for S1R binding, the isolated domain was titrated against S1R(cd). Addition of the NBD resulted in similar changes in intensities as full-length BiP (Fig. 8*B*), indicating that the BiP NBD was sufficient for S1R interactions. The NBD domain was generally less stable than full-length BiP and more unstable in the presence of calcium, preventing evaluation of the effects of calcium on the NBD interaction with S1R(cd). In contrast, no changes in intensities or chemical shifts were observed after addition of the SBD (Fig. 8*D*).

## DISCUSSION

Most membrane-associated proteins remain difficult to express at sufficient levels to enable inexpensive isotope labeling. We have found that the chaperone domain of human S1R could be efficiently expressed in *Escherichia coli* using the single protein production approach (23). In single protein production, the cells remain metabolically active and express the target protein, but cell growth is halted, which appears to greatly diminish expression toxicity. The approach permitted 10-fold condensation of the cultures when transferring the cells into labeled medium and resulted in yields of 8–10 mg of pure S1R(cd) per liter of labeled medium.

The S1R chaperone domain is predicted to contain a C-terminal membrane-associated region (residues ∼180–203). Consistent with this prediction, solubilization of S1R(cd) required the presence of lipid or detergent, suggesting that membrane interactions may be required also for proper folding of S1R(cd) *in vivo*.

For the NMR studies reported here, S1R(cd) was reconstituted from 8 m urea into DPC, which contains a phosphocholine headgroup and has been used extensively for studying folded integral membrane and membrane-associated proteins (28–31). The S1R(cd) samples permitted measurement of high quality NMR spectra, and near UV CD indicated the presence of temperature-sensitive tertiary structure. The finding that the S1R(cd) construct purified from *E. coli* and reconstituted with DPC micelles interacted with BiP in a calcium-dependent manner indicated that S1R(cd) adopted a native-like conformation in the NMR conditions, at least in respect to those elements required for the BiP interaction.

The N-terminal half of S1R(cd) contains a ∼17-residue helix, and at least two short β-strands. The region containing the extended regions, residues ∼140–160, is highly dynamic, based on secondary chemical shifts, relaxation measurements, and amide proton exchange rates.

The putative membrane associated C-terminal half of S1R(cd) is largely helical, with four helices separated by short, flexible, water-exposed linkers. By combining information from NOEs to detergent and resonance broadening from a water-soluble paramagnetic agent, a stretch of ∼9 amino acids (residues 198–206) within helix 4 was identified as a site for strong interactions with the detergent micelle. Although helix 4 is ∼18 residues long, it is not highly hydrophobic (containing two arginines and an aspartic acid), and residues 196, 197 and 207 have detectable rates of amide proton exchange with water. This suggests that it is not entirely embedded within the detergent micelle as could be expected for a transmembrane helix. Instead, residues 198–206 form an amphipathic helix, which likely interacts with the surface of the detergent micelle, and is therefore also a likely site for attachment to the ER membrane.

A cluster of NOEs to detergent were also observed in residues ∼183–189, suggesting that this region may also facilitate micelle or membrane binding. However, these residues are also largely water-accessible and are proposed to have long-range intramolecular interactions in the intact receptor (35). Thus, it appears unlikely that the chaperone domain contains a helix capable of spanning the ER membrane. The lack of a transmembrane helix in the chaperone domain is consistent with the finding that this domain can be extracted from cells with chaotropic salt washes (14).

**FIGURE 9.**
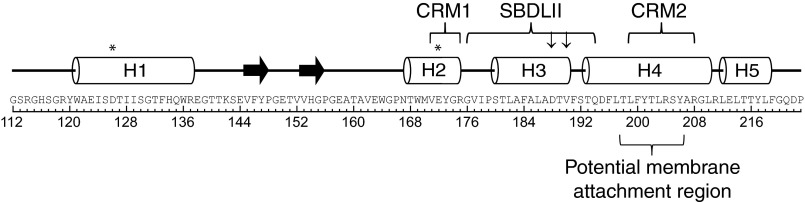
**The helical (*cylinders*) and extended (*arrow*) residues of S1R(cd) determined from a combination of chemical shifts, amide-water proton exchange, ^15^N relaxation rates, and NOEs.** Residues and regions previously implicated in cholesterol binding are indicated: cholesterol binding motifs (CRM1 and CRM2), cocaine binding (SBDLII; residues Asp-188 and Val-190 are indicated by ↓), and haloperidol binding (residues Asp-126 and Glu-172 are indicated by an *asterisk*). Residues 198–206, which are proposed to have the strongest interactions with the ER membrane, are indicated. The amino acid sequence and corresponding residue numbers are shown at the *bottom*.

The chaperone domain contains several regions implicated in cholesterol and drug interactions (Fig. 9). Based on a cholesterol recognition consensus sequence (32, 33) (L/V)*X*_1–5_Y*X*_1–5_(R/K) two cholesterol recognition motifs have been identified within the structured region of S1R(cd) (17, 34, 35). The first motif (CRM1; residues 171–175; amino acid sequence, VEYGR) corresponds approximately to helix 2. The second motif (CRM2; residues 199–208; amino acid sequence, LFYTLRSYAR) contains two overlapping cholesterol recognition motifs (Leu-199/Tyr-201/Arg-204 and Leu-203/Tyr-206/Arg-208), and corresponds to the central region of helix 4. Residues in the vicinity of both CRM1 and CRM2 exhibited heightened protection from the water-soluble paramagnetic agent, although a larger number of NOEs to DPC were detected in the CRM2, suggesting a more intimate interaction with the detergent micelle at this site.

Although the chaperone domain does not bind drugs in the absence of the N-terminal transmembrane domain (1, 35), the secondary structure determined here may reflect the structural propensities of the intact receptor. Studies using a chemically reactive affinity probe have provided information on the binding site of cocaine in guinea pig S1R, which is 98% similar to human S1R (35–37). Those studies defined a steroid binding-like domain (SBDLII) at residues 176–194 and located residues Asp-188 and Val-190 close to the cocaine interaction site. SBDLII together with a steroid binding-like domain (denoted SBDLI; residues 91–109) in the second putative transmembrane helix stabilizes cocaine binding (35). SBDLII corresponds closely to helix 3 (residues 180–189) and the adjacent residues connecting helix 3 to helices 2 and 4 (Fig. 9). Asp-188 and Val-190, which are proposed to be close to the cocaine binding site, are at the C-terminal end of helix 3. Both Asp-188 and Val-190 are solvent exposed in S1R(cd), and Val-190 is one of the most flexible residues within the C-terminal helical region of S1R(cd). It is unknown whether the flexibility observed here is preserved in full-length S1R, but such flexibility may facilitate binding-induced conformational changes necessary to mediate S1R downstream interactions.

Mutational studies of S1R have also implicated Asp-126 and Glu-172 in haloperidol binding (8). Asp-126 and Glu-172 are found in helices 1 and 2 of S1R(cd), respectively, suggesting that these helices may interact in the haloperidol bound conformation of the receptor.

BiP is an ER resident chaperone that regulates the unfolded protein response in addition to assisting protein folding (reviewed in Ref. 38). S1R is proposed to sequester BiP in the absence of ER stress. Upon depletion of ER calcium or S1R ligand binding the S1R·BiP complex dissociates leading to chaperone activity and downstream signaling of S1R, including inositol triphosphate receptor-mediated calcium release (1, 3). We have shown here that S1R(cd) under NMR conditions interacts with BiP in a calcium-dependent manner and that the NBD of BiP is sufficient for these interactions. The finding that S1R(cd) interacts with the NBD of BiP is consistent with the known regulatory interactions with other Hsp proteins. For example, the co-chaperone BAG1 binds to the NBD of Hsc70 (42, 43), and bacterial GrpE binds to the NBD of DnaK (44, 45).

The role of the region ∼140–160 remains unknown. This region did not appear to tightly associate with BiP in our studies, and substitution of acidic residues within this region has been shown to have no impact on haloperidol binding (8). Based on sequence analysis (39), several short β-strands are predicted for this region in residues 143–145, 151–153, and 159–164, which corresponds approximately to those regions observed to be extended here (residues 145–147, 153–155; secondary chemical shifts also indicate transient extended conformation in residues 160–162). However, sequence analysis also predicts a high degree of disorder in this region (40), which is confirmed by the NMR data. By contrast, residues 124–137 and 168–173 are predicted from sequence to be extended but found here to be helical. DPC has previously been shown to disrupt β-sheets in a concentration-dependent manner (41). However, the concentrations used here are as much as 10-fold lower than in that study, and the spectra of S1R(cd) in 5 and 30 mm DPC are essentially identical.

In summary, we have studied the chaperone domain of S1R by solution NMR and have characterized its secondary structure and dynamics. S1R(cd) is composed of five helices and at least two short extended regions in a dynamic region between helices 1 and 2. Three of the helices in the C-terminal membrane-associated region map to residues previously identified as important in cholesterol and cocaine binding. A fourth helix (helix 4) is implicated in membrane association. In addition, we have shown that the NBD domain of BiP is sufficient for interaction with the S1R chaperone domain. These results advance our understanding of S1R and are likely to be useful in refining models of the S1R drug binding sites (46). Future studies are needed to identify tertiary interactions in the S1R chaperone domain and to further delineate S1R/BiP and S1R ligand and cholesterol interactions.

## Footnotes

3 The abbreviations used are:

S1R: sigma-1 receptor
S1R(cd): sigma-1 receptor chaperone domain
ER: endoplasmic reticulum
BiP: binding immunoglobulin protein
DPC: dodecylphosphocholine
NBD: nucleotide binding domain
SBD: substrate binding domain
SBDL: steroid binding-like domain
CRM: cholesterol recognition motif.
